# Individual behavioral correlates of tail biting in pre-finishing piglets

**DOI:** 10.3389/fvets.2022.1033463

**Published:** 2022-12-05

**Authors:** Marc Bagaria, Laura Kuiper, Ellen Meijer, Elisabeth H. M. Sterck

**Affiliations:** ^1^Animal Behavior & Cognition, Utrecht University, Utrecht, Netherlands; ^2^Animal Welfare Program, Institute of Agrifood Research and Technology (IRTA), Monells, Spain; ^3^Population Health, Veterinary Sciences, Utrecht University, Utrecht, Netherlands; ^4^Animal Science Department, Biomedical Primate Research Centre, Rijswijk, Netherlands

**Keywords:** pigs, tail biting, tail docking, two-stage, sudden-forceful, behavior, social

## Abstract

**Introduction:**

Tail biting is a widespread problem in pig production systems and has a negative impact on both animal welfare and farm income. This explorative study aims to validate how tail biting is related to general behaviors at the individual level and explore whether these behaviors are related to a particular type of tail biting: two-stage, sudden-forceful, obsessive, or epidemic.

**Methods:**

This research was conducted in a standard commercial setting where 89 tail-docked pre-finishing piglets divided into 8 groups were observed 4 days per week from 5 to 8 weeks of age. Each piglet was observed for a total of 160 min using continuous focal sampling. Ten individual behaviors were recorded based on the general behaviors expected to be linked to giving tail biting (PCA1), receiving tail biting (PCA2), and tail biting damage (PCA3). These PCAs were assembled and related to tail biting given, tail biting received, and tail biting lesions.

**Results:**

Tail biting did not lead to major damage on the piglets' tail at 8 weeks of age but was observed 420 times, where most of the individuals (72%) were categorized as “biters and victims.” When relating PCA1 with tail biting given, piglets that gave more tail biting showed more “active exploration.” When relating PCA2 with tail biting received, piglets receiving more tail biting were more “explored while active” and “attacked and explored.” When relating PCA2 with tail biting lesions, piglets presenting lesions showed more “agonism.” Surprisingly, tail biting lesions were not significantly related to PCA3. The relationship between explorative behaviors and tail biting indicates that the pre-damage stage of two-stage tail biting was the predominant tail biting type, while the damaging stage was likely incipient. The relationship between tail biting and aggression, as well as the minor tail lesions observed suggest that sudden-forceful tail biting was probably present even though it was rarely seen. Obsessive and epidemic tail biting were not observed.

**Discussion:**

This study demonstrates that studying tail biting at the individual level helps to identify the type of tail biting present. This gives directions to farmers for applying appropriate measures to prevent the development of tail biting behavior in piglets.

## Introduction

Tail biting is a recurrent behavioral issue in pig production systems. This behavior can lead to tail damage, which is problematic from both a welfare and an economical perspective (1). The incidence of tail biting can be reduced by tail docking, a process that involves amputating the distal part of the pig's tail (2). However, this widespread farming practice does not eliminate the tail damage entirely: (3) studies suggest that tail lesions occur in approximately 1–3.1% of tail docked pigs (4, 5). In addition, tail docking has negative welfare consequences for the animals, including acute stress and acute and chronic pain (6). The Directive 2008/120/EC (7) states that: “*Before carrying out tail-docking other measures are to be taken to prevent tail-biting and other vices, taking into account environment and stocking densities. For that reason, inadequate environmental conditions or management systems are to be changed.”* Indeed, while tail docking can be useful in preventing symptoms of tail biting behavior, it does not eliminate its causes (8).

The occurrence of tail biting behavior is rather unpredictable as its motivations and underlying causes remain unknown (9). Tail biting has a multifactorial background, as its risk factors appear to be related to both environmental factors and the biological characteristics of individual pigs (9). These risk factors can increase the stress levels of the animals, which in turn may influence a broad spectrum of motivational systems including those regulating explorative behavior, social behavior, foraging, and thermoregulation (10). Severe and persistent stress can create a pre-pathological state in pigs that ultimately decreases reproduction and changes metabolism. Moreover, it may lead to the development of detrimental behaviors such as tail biting (11).

Consistent with the wide variety of behavioral patterns related to tail biting, four types of tail biting have been proposed: two-stage, sudden-forceful, obsessive (12), and epidemic tail biting (10). These four different types of tail-biting may be related to different behaviors. First, two-stage tail biting is made up of a pre-damage stage and a damaging stage. Pre-damage is where a pig takes the tail of another pig in its mouth, manipulating it without causing visible damage or a reaction from the bitten pig. At some point, the manipulation may break the skin of the tail and cause bleeding, forming the start of the damaging stage. The damaging stage is characterized by a damaged tail with minor scratches or severe wounds and an avoidance reaction from the bitten pig. Two-stage tail biting seems to be caused by a lack of chewing and manipulation objects or substrates needed to satisfy the foraging and exploratory needs of the pigs (13, 14). Two-stage tail biting can be prevented by the provision of appropriate manipulatable objects or substrates (12). Second, sudden-forceful tail biting is an aggressive behavior wherein a pig grabs and yanks the tail of another pig, causing a reaction from the victim and an injury to its tail. Sudden-forceful tail biting is considered an aggressive frustrated behavior caused by inadequate access to resources (15). This type of tail biting can be prevented by decreasing competition between individuals and increasing the availability of resources, such as food, water, or enrichment (16). Third, obsessive tail biting occurs when a pig repeatedly grabs and yanks the tail of another pig, causing a reaction from the victim. In this type of tail biting, the biter is fixated on performing this behavior and persistently looks for tails to bite. The cause of obsessive tail biting is unknown, though it seems to be related to the biological characteristics of the pig. The risk of obsessive tail biting can be reduced by identifying pigs with obsessive tendencies and removing them from the pen (17). More recently, a fourth tail biting type, “epidemic tail biting,” has been identified (10). This is a sudden event that results in mild to severe tail damage that spreads quickly through a single or multiple pens. Acute changes such as feeding problems or changes in temperature can cause epidemic tail biting. The risk of epidemic tail biting can be minimized by managing farms in a way that prevents sudden changes (10). As each type of tail biting is related to a different set of motivations, they each require their own set of interventions (12). Thus, identifying a specific tail biting type can provide an indication of which preventive action(s) to take.

Tail biting is typically described at group level, wherein tail biting outbreaks are characterized as the stage in which there is an increasing number of bleeding tails injuries caused by tail biting behavior (18). Most studies aim to identify behavioral changes prior to the onset of an outbreak in order to predict them. These studies found that in pens where tail biting damage was detected, pigs were more active (9, 19, 20), pen and pen mates' directed manipulations were higher (9), and object manipulation (19) and tail interest were lower (20). Other studies that aimed to characterize tail biters and victims at the individual level found that tail biters performed more ear biting, mounting (21), sniffing (22), manipulative behaviors directed toward the pen, enrichment (23), and pen mates' tail (21, 23), and received more aggression (23). Tail biting victims received more tail manipulation (21, 23) and sniffing (22), were more aggressive, and changed posture more frequently (23). However, these studies were mostly conducted on groups of undocked finishing pigs where tail biting damage was already present (20–22, 24). Tail biting outbreaks usually appear during the finishing phase and are not common before the ages of 90 to 130 days (2, 25). Despite this, tail damage may appear in the pre-finishing phase as soon as piglets are weaned (26), and precursors of tail biting behavior appear even earlier, during the suckling period (9, 27). Yet only a few studies conducted observations during this time period (9, 22, 28) and they did not classify individual pigs as showing one of the four types of tail biting. Studying tail biting at its origin and classifying it may be critical to establishing proper advisory measures for individual farms (29).

The main objective of this explorative study was to validate how tail biting is related to general behaviors at the individual level in pre-finishing piglets. In addition, we discuss whether these behaviors were related to particular types of tail biting. In the present study, a distinction was made between the receivers of tail biting (victims) and those performing tail biting (biters). The tail lesions provoked by tail biting were also studied. This research was conducted on 8 groups of weaned piglets housed in a standard commercial setting. Predictions were made based on general behaviors that have been related to giving tail biting, receiving tail biting, and tail damage in previous studies. Firstly, we expected piglets that performed more tail biting to engage in more explorative behaviors toward the pen and their pen mates, including tail exploration, receive more aggression, and being more active. Secondly, we expected the behavior “receiving tail biting” to be positively correlated to restlessness, giving aggression, and receiving exploration from pen mates, particularly to the tail. Thirdly, we expected that the presence of lesions on a pig's tail would be positively correlated to their level of activity, as well as the amount of manipulation they directed toward their pen and pen mates, and negatively correlated to their levels of tail interest and object manipulation.

Lastly, we aimed to use the relationship between tail biting and general behaviors to identify the type(s) of tail biting present in the studied animals. Based on the different motivations of each tail biting type, we expected to encounter two-stage tail biting if tail biting was related to explorative behaviors directed toward the pen and pen mates. We expected visible tail damage and reactions from the victims to be absent if the pre-damage stage was occurring and present if the damaging stage was occurring. We expected to encounter sudden-forceful tail biting if tail biting was related to aggressive behaviors and there was a reaction from the victim and visible tail damage was present. Obsessive tail biting was not expected to have a link to any particular behavior but was expected to be encountered if tail biting was performed persistently by a single or few individual(s), there was a reaction from the victim and visible tail damage was present. Epidemic tail biting was not expected to have a link to any particular behavior but was expected to be encountered if it appeared unexpectedly in one or more pens causing mild to severe tail damage to several piglets.

## Materials and methods

### Ethical statement

All methods that demanded the handling of live animals were reviewed and approved by the local animal welfare body (Animal Welfare Body, Utrecht University). Since this study aimed to study tail biting in a real conventional context, only observational measures were taken, and the treatment of the animals followed the usual management procedures of the farm. Thus, the Animal Welfare Body did not consider this an animal experiment and it was performed without any further permission.

### Animals and housing

**Figure 1:**
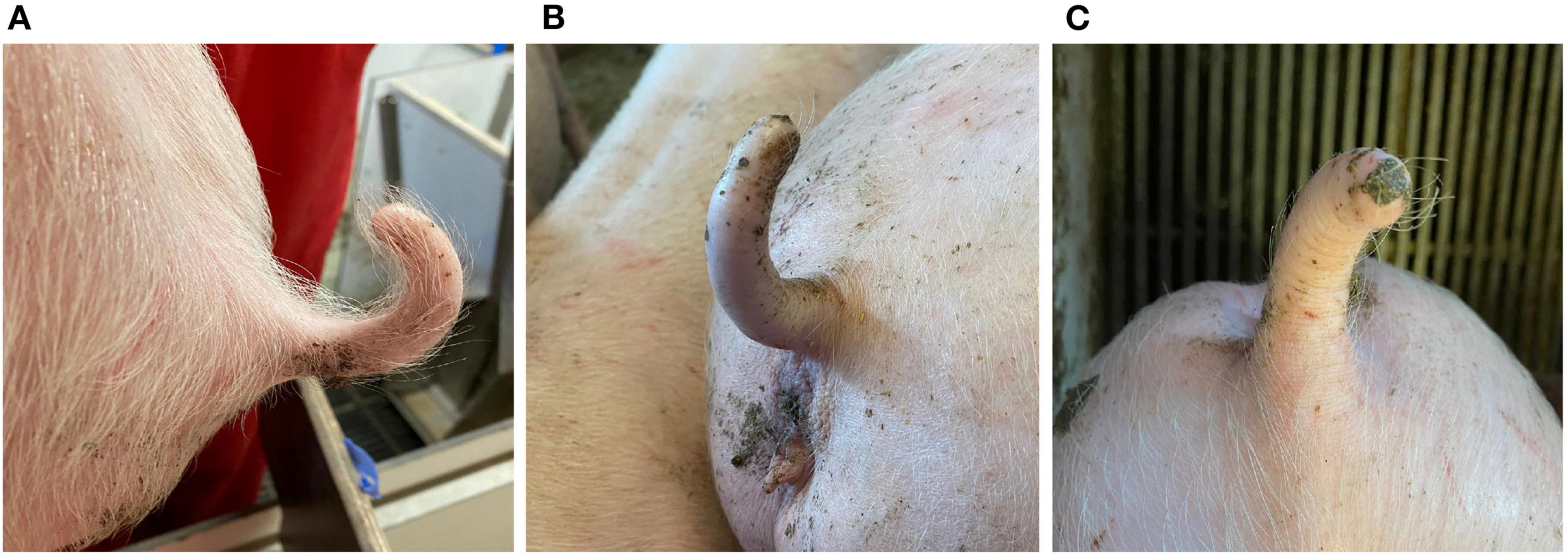
Tail lesions scores: **(A)** no damage (category 1), **(B)** damage; no hair (category 2), and **(C)** damage; minor scratches (category 3).

The study was conducted at De Tolakker, the farm of the Faculty of Veterinary Medicine of Utrecht University, Utrecht, the Netherlands. The farmers reported that tail lesions provoked by tail biting were seen persistently at the farm during the pigs' rearing phase, even though tail docking was performed routinely. The farm followed standard commercial procedures and consisted of 120 *Norwegian Landrace x Yorkshire* sows. Piglets were provided an analgesic and tail docked using a heated clipper at the age of 4 days, leaving a long-docked tail with approximately 2.5 cm of length (Figure 1). Castration and teeth clipping were not performed on the farm and piglets were ear-tagged for individual identification before weaning. Piglets were weaned when they were separated from the sow at 28–29 days old and moved to a new pre-finishing pen where four unmixed (both sexes from the same litter) and four mixed (only male piglets from more than one litter) groups of 10 to 13 individuals were formed. Groups were formed according to the farm usual management procedures, with no intervention from the observers. Weaned piglets were housed in pens 3.70×1.35 m (length x width) that had a concrete and partially slatted iron floor. Each pen contained a 0.66 x 0.30 m feeder with *ad libitum* dry pellets, an *ad libitum* water supply, and two enrichment locations made up of one hanging metal chain and one rubber bite toy. The weaned piglets stayed in these pens from weaning until their transport to the fattening farm at 66 days of age. The temperature of the room was maintained between 18°C and 24°C by a ventilated heating system.

### Behavioral observations

A complete ethogram was compiled based on an extensive literature review prior to conducting observations. The ethogram used in this study included 10 general behaviors that were expected to be related to tail biting behavior based on this literature. The general behaviors identified were tail manipulation with reaction, tail manipulation without reaction, tail exploration, ear manipulation, environment exploration, lying, sitting, standing, nosing, and aggression (Table 1) (9, 20, 30–32).

**Table 1:** Ethogram of the behaviors included in this study for the focal behavioral observations.

**Behavior**	**Description**	**Focal sampling method**
Tail manipulation with reaction	Putting the tail of another pig in their mouth while biting or pulling hard enough to cause a reaction in the bitten pig (20).	Event
Tail manipulation without reaction	Putting the tail of another pig in their mouth without biting or pulling hard enough to cause a reaction in the bitten pig (20).	Event
Tail exploration	Sniffing, nosing, or manipulating the tail of another pig without taking the tail into their mouth (20).	Event
Ear manipulation	Biting, sniffing, nosing, or manipulating the ear of another pig with or without taking the ear into their mouth (20).	Event
Environment exploration	Rooting, pawing, nosing, chewing, or licking an enrichment object or any part of the pen that is not an enrichment object (30).	State
Lying	Lying laterally on the side or ventrally on the sternum (31)	State
Sitting	The body is supported by two bent back or front legs while the other two legs are straight (31).	State
Standing	Standing with four straight legs on the floor (31).	State
Nosing	The nose of the pig approaches the nose, head, ears, body, or genitals of another pig and there is at least a short duration of physical contact (32).	Event
Aggression	A pig is aggressively chasing, replacing, pressing, levering, knocking, or biting a pen mate (32).	Event

Continuous focal sampling, in which the animal of interest (the focal) was observed during different observation periods, was used for the behavioral observations (33). Live observations were conducted on the farm and scored using a Tablet and the Behavioral Observation Research Interactive Software (BORIS) (34). During an observation period, all behaviors performed by the focal animal and all directional behaviors performed by other individuals toward the focal animal were scored. Point behaviors (events) are frequency-based and scored every time they occurred. These included tail manipulation with reaction, tail manipulation without reaction, tail exploration, ear manipulation, nosing, and aggression. A new occasion of these behaviors was scored when the focal did not perform the behavior for 5 seconds. Duration behaviors (states) are mutually exclusive and were scored according to the amount of time the animal spent performing the behavior, which included environment exploration, lying, sitting, and standing (Table 1) (33).

During the observation period, a total of 89 individuals were divided into 8 groups and observed in two rounds. The first round started on the 17^th^ of August 2020 and lasted until the 11^th^ of September, and the second round started on the 14^th^ of September and lasted until the 9^th^ of October. During the first round, four unmixed groups were observed, each containing 12–13 male and female piglets. During the second round, four male-only mixed groups of 10 piglets were observed. After being weaned at 4 weeks of age, piglets were acclimated to the new environment and each group was observed for four consecutive weeks from 5 weeks of age until 8 weeks of age. For individual recognition, each piglet wore an ear tag with an individual number code and was marked with a unique color-symbol combination using a semi-permanent spray. Observations were conducted Monday's through Thursday's each week, observing each individual once in the morning and once in the afternoon for 5 min each time. By the end of the observation period, each individual was observed for a cumulative total of 160 min. Observations were performed between the hours of 9:00 and 16:00, alternating the order in which each group was observed throughout the day to counterbalance the observation times over the 4 days. The measures of behavior were expressed as number of times (events) or duration (states) an individual performed the behavior during the entire observation period. The observations were conducted by two observers (MB and LK) whose inter-observer reliability (IOR) was calculated prior to the start of the observation period using a Kappa Coefficient test. The result of the Kappa Coefficient test was 0.74, which indicated an acceptable IOR (33). Both observers discussed the disagreements and proceeded with the behavioral observations.

### Measures of tail biting

Three types of tail biting measures were used in this study: tail biting given; tail biting received; and tail biting lesions. In the ethogram, a distinction was made between “tail manipulation with reaction” and “tail manipulation without reaction” but due to the low incidence of the first behavior, they were summed and analyzed as “tail biting” behavior. The measure of tail biting given was based on the number of times “tail biting” behavior was performed, and “tail biting received” on the number of times “tail biting” behavior was received. To identify the number of individuals acting as biters and/or victims, individuals that only gave tail biting at least once during the observation period were classified as “biters” and individuals that only received tail biting at least once during the observation period were classified as “victims.” The number of individuals that both performed and received tail biting at least once during the observation period were classified as “biters and victims” and the number of individuals that neither performed nor received tail biting during the observation period were classified as “neutrals.”

**Figure 2:**
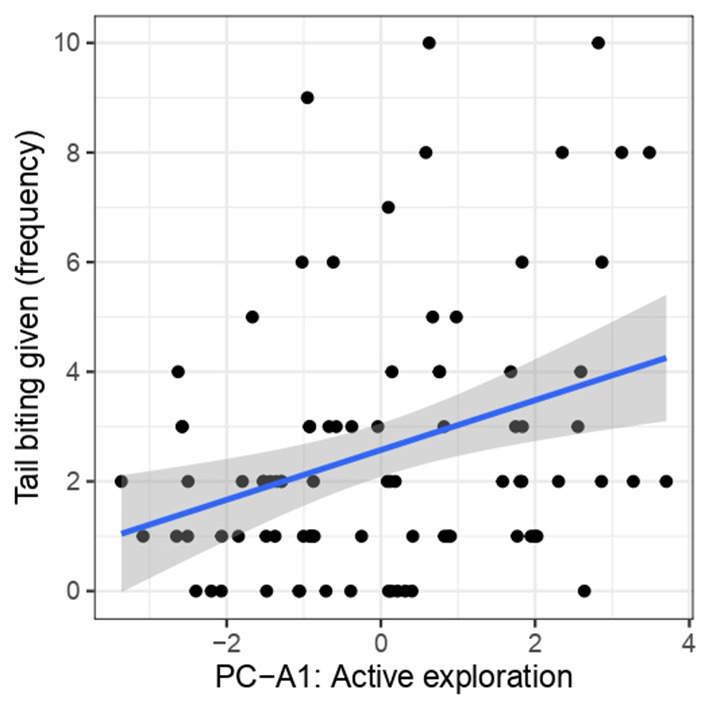
The relationship between tail biting given and PC-A1 “Active exploration.”

**Figure 3:**
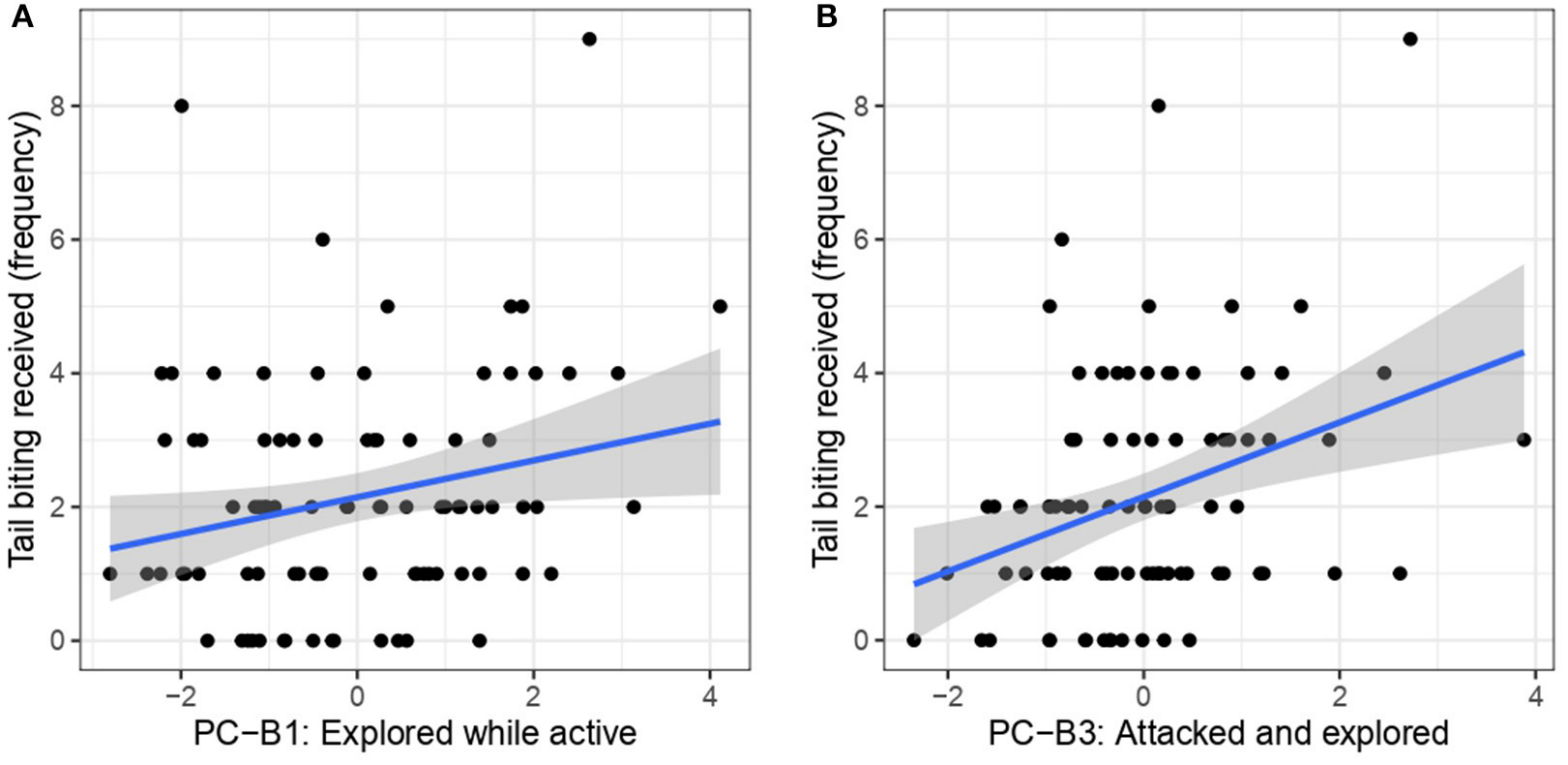
The relationship of tail biting received to PC-B1 “Explored while active” **(A)** and PC-B3 “Attacked and explored” **(B)**.

**Figure 4:**
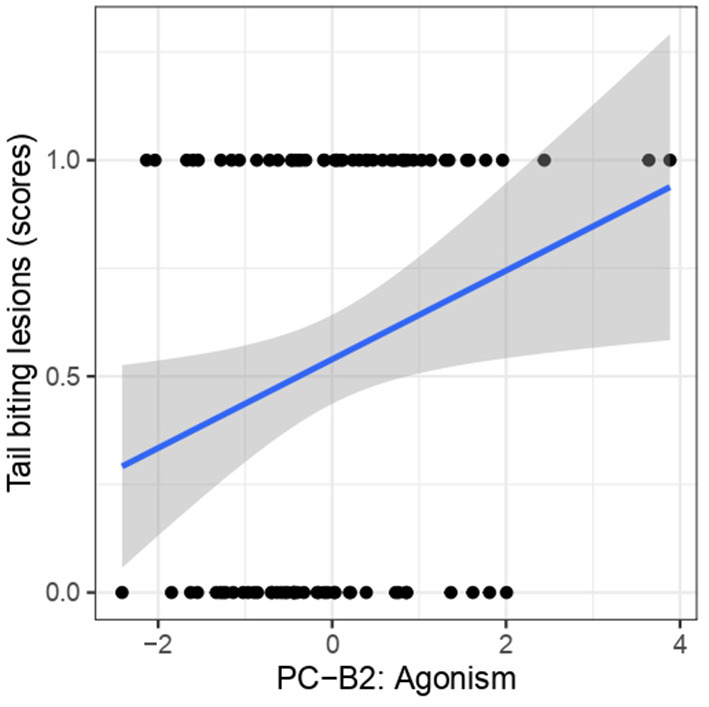
The relationship betwen tail biting lesions scores and PC-B2 “Agonism.”

At the end of each observation week (weeks 5 through 8), the presence or absence of lesions on the tail of each piglet were recorded using the following categories: (1) no damage (no visible tail lesions; hair present) (Figure 1A), (2) no hair (no visible tail lesions; hair not present) (Figure 1B), (3) minor scratches (superficial scratches) (Figure 1C), (4) wound (visible wound and tissue damage), and (5) severe wound (the outer part of the tail has almost been bitten off and the length of the tail is reduced) (28). A tail biting outbreak was identified when one or more pigs within a pen showed tail lesions in the category of wound or severe wound (28). Since we did not find any tail damage (category 2 or higher) up to observation week 7, we do not report these results. Thus, the tail biting lesions were the scores of the last day of observations on week 8. Given the low frequency and severity of damage observed (categories 4 and 5 were not observed), tail lesions were redefined as a binomial variable and categorized as either no damage (category 1) = 0, or damage (no hair, minor scratches, category 2 or 3) = 1. These values were entered in statistical analyses.

### Statistical analysis

The software Rstudio v.1.3.1093 (35) was used to conduct the statistical analysis. Tests were two-sided with an alpha value of 0.05. When normal distribution was assumed, it was checked creating a histogram and using a Shapiro-Wilk test. If needed, data were transformed using a logarithmic transformation until Gaussian distribution was met.

Firstly, the relationship between the three tail biting measures (given, received, and lesions) were analyzed. To investigate the relationship between tail biting given and received, a linear mixed model (LMM) was used. It included tail biting given as response variable, tail biting received as a fixed factor, and a random intercept for group nested within the fixed effect group formation (mixed or unmixed) (Model 1). To investigate the relationship between tail biting lesions and tail biting given or received, a generalized linear mixed model (GLMM) with a binomial distribution was used. It included tail biting lesions as response variable, tail biting given and tail biting received as fixed factors, and a random intercept for group nested within the fixed effect group formation (mixed or unmixed) (Model 2).

Secondly, three different PCAs (Principal Component Analysis) were run using general behaviors predicted to be related to giving tail biting (PCA-A), receiving tail biting (PCA-B), or tail biting damage (PCA-C) in order to determine if any of the included behaviors loaded on the same PC-axis (Principal Component). Three different PCAs were conducted due to different general behaviors that are related to the behaviors giving tail biting, receiving it, or presenting tail damage. The behaviors included on each PCA were extracted from the outcomes of previous studies that related tail biting to general behavior. After running the PCAs, the Kaiser criterion was followed, the PCs with eigenvalues >1 were retained (36) and their loading values were calculated. Following classical heuristics, the interpretable loadings were those with values ≥0.4 (N individuals = 89) (37). Once these interpretable behaviors were identified, each of the PCs was assigned a descriptive label indicating a behavioral category that summarized the loaded behaviors. Lastly, the individual component scores of the PCs were obtained using the regression method.

To study the relationship between the general behaviors summarized in the PCAs and tail biting given, tail biting received, and tail biting lesions, different LMM and GLMM models were used (Models 3 to 6) that contained the PCs of one of the three PCAs (A, B, and C). To investigate the relationship between tail biting given and tail biting received with the general behaviors that were predictor variables for giving tail biting (PCA-A) and receiving tail biting (PCA-B), two LMM were used. They included tail biting given (Model 3) or tail biting received (Model 4) as the response variable, the included PCs (PCA-A or PCA-B, respectively) as fixed factors, and a random intercept for group nested within the fixed effect group formation (mixed or unmixed). To investigate the relationship between tail biting lesions with the general behaviors that were predictor variables for receiving tail biting (PCA-B) and tail biting damage (PCA-C), two GLMM with a binomial distribution were used. They included tail biting lesions as the response variable, the included PCs [PCA-B (Model 5) or PCA-C (Model 6)] as fixed factors, group as a random effect, and a random intercept for group nested within the fixed effect group formation (mixed or unmixed).

For each of the 6 models, a comparison was made between the full model and a reduced model without the random effects. Following the Akaike's Information Criterion (AIC), the model with the lowest AIC values was selected (38) to identify the best fitting model. The reduced models excluding the random effects (group and group formation) were selected in each case, thus these are the presented in the results. Results were obtained by running the best fitting LMM using a type III ANOVA test and GLMM using a chi-square test.

## Results

### Tail biting measures

“Tail biting” behavior, which is the sum of “tail manipulation with reaction” and “tail manipulation without reaction” behaviors, was observed a total of 420 times during this study: “tail manipulation without reaction” was observed 387 times (92%) and “tail manipulation with reaction” was observed 33 times (8%). The behavior “tail manipulation without reaction” was performed by 74 individuals (83%) and received by 71 (80%) while “tail manipulation with reaction” was performed by 8 individuals (9%) and received by 16 (18%).

Of the 89 individuals in the study, 12 individuals (14%) only gave tail biting and were categorized as “biters,” while 11 individuals (12%) only received tail biting and were categorized as “victims.” 64 individuals (72%) gave and received tail biting and were categorized as “biters and victims,” while 2 individuals (2%) neither gave or received tail biting and were categorized as “neutrals.” Of the 89 piglets, 13 (15%) did not give tail biting and 14 (16%) did not receive it during the observation period, 76 (85%) gave between 1 and 10 tail bites, and 75 (84%) received between 1 and 9 tail bites. It was noted by the observers that at the time of tail biting, both the victim and the biter were usually lying down.

None of the studied animals presented tail lesions (scores 2 or higher) before 8 weeks of age. On the last day of observations, at the end of week 8, the following scores were found for each tail lesions category: (1) no damage, *N* = 41 piglets, 46%, (2) no hair, *N* = 39 piglets, 44%, and (3) minor scratches, *N* = 9 piglets, 10%. None of the piglets had a wound (4) or severe wound (5).

When assessing how the three measures of tail biting were related, the results of Model 1 demonstrated that tail biting given and received were not significantly related (F1, 87 = 0.61, *p* = 0.44) (Annex 1). The results of Model 2 demonstrated that both tail biting given [X^2^ = 1.83, *p* = 0.19; OR = 1.13, 95% CI [0.95, 1.37]] and tail biting received [X^2^= 0.30, *p* = 0.58; OR = 0.93, 95% CI [0.73, 1.19]] had no significant relationship with the scores of tail lesions (Annex 2).

### Principal component analysis

The general behaviors were related in a separate PCA for each of the following: giving tail biting (PCA-A), receiving tail biting (PCA-B), and tail biting damage (PCA-C).

In the PCA-A, where predictor variables for giving tail biting were analyzed, 3 PCs that had eigenvalue >1 and accounted for 57.9% of the variance were included. PC-A1 was positively related to explorative behaviors, enhanced activity, and also aggression of the animals, thus labeled as “active exploration.” PC-A2 was positively related to giving aggression, receiving aggression, and sitting, thus labeled as “fighting.” PC-A3 was positively related to ear manipulation, environment exploration, and sitting, thus labeled as “ear-directed manipulation” (Table 2: PCA-A).

**Table 2:** Results of the PCAs for giving tail biting (PCA-A), receiving tail biting (PCA-B), and tail biting damage (PCA-C).

**PCA-A: Giving tail biting**	**PC-A1: Active exploration**	**PC-A2: Fighting**	**PC-A3: Ear-directed manipulation**	**PC-A4 (not included)**
**Eigenvalue; variance percent**	**2.84; 31.5%**	**1.26; 14.0%**	**1.11; 12.3%**	**0.92; 10.3%**
Aggression	0.40	0.61	-	-
Receiving aggression	-	0.60	-	-
Tail exploration	0.52	-	-	-
Ear manipulation	-	-	0.46	-
Nosing	0.64	-	-	-
Lying	−0.70	-	-	-
Sitting	-	0.52	0.70	-
Standing	0.88	-	-	-
Environment exploration	0.71	-	0.42	-
**PCA-B: Receiving tail biting**	**PC-B1: explored while active**	**PC-B2: Agonism**	**PC-B3: Attacked and explored**	**PC-B4 (not included)**
**Eigenvalue; variance percent**	**2.08; 26.1%**	**1.39; 17.4%**	**1.14; 14.2%**	**0.98; 12.2%**
Aggression	-	0.59	-	-
Receiving aggression	-	0.56	0.41	-
Receiving tail exploration	0.42	-	0.43	-
Receiving ear manipulation	-	-	0.81	-
Receiving nosing	0.47	−0.57	-	
Lying	−0.76	-	-	-
Sitting	−0.46	0.47	-	-
Standing	0.84	-	-	-
**PCA-C: Tail biting damage**	**PC-C1: Active pen and tail exploration**	**PC-C2: Non-social exploration**	**PC-C3: Ear manipulated and tail exploration**	**PC-C4: attacked and not tail explored**
**Eigenvalue; variance percent**	**2.53; 28.1%**	**1.39; 15.5%**	**1.20; 13.3%**	**1.04; 11.5%**
Receiving aggression	-	-	-	0.62
Tail exploration	0.47	-	0.43	-
Receiving tail exploration	0.42	-	-	−0.53
Receiving ear manipulation	-	-	0.83	-
Receiving nosing	-	−0.74	-	-
Lying	−0.74	-	-	-
Sitting	-	0.67	-	−0.41
Standing	0.90	-	-	-
Environment exploration	0.72	0.41	-	-

Each PCA shows the PCs (eigenvalue ≥ 1) included along with their assigned label, interpretable loadings (≥ 0.4), eigenvalues, and variance percentage. The loadings with a value < 0.4 are not presented in the table.

In the PCA-B, where the predictor variables for receiving tail biting were analyzed, 3 PCs that accounted for 57.8% of the variance were included. PC-B1 was related to receiving exploration and enhanced activity of the animals, thus labeled as “explored while active.” PC-B2 was positively related to giving aggression and receiving aggression, and negatively related to receiving nosing and sitting, thus labeled as “agonism.” PC-B3 was positively related to receiving aggression and receiving tail and ear manipulation, thus labeled “attacked and explored” (Table 2: PCA-B).

In the PCA-C, where the predictor variables for tail biting damage were analyzed, 4 PCs that accounted for 68.5% of the variance were included. PC-C1 was positively related to environment exploration, giving tail exploration, receiving tail exploration, and enhanced activity of the animals, thus labeled as “active pen and tail exploration.” PC-C2 was positively related to environment exploration and sitting, and negatively related to receiving nosing, thus labeled as “non-social exploration.” PC-C3 was positively related to receiving ear manipulation and performing tail exploration, thus labeled as “ear manipulated and tail exploration.” PC-C4 was positively related to receiving aggression and negatively related to tail exploration and sitting, thus labeled as “attacked and not tail explored” (Table 2: PCA-C).

### The relationship between PCs and tail biting measures

The PCs of general behaviors related to a particular tail biting behavior were entered as behavioral estimates in the respective Models 3–6.

When analyzing the relationship between PCA-A and tail biting given, the results of Model 3 showed that individuals expressing more “active exploration” (PC-A1) had a significantly higher frequency of tail biting given (F1, 85 = 8.41, *p* = 0.004) (Figure 2). There was no significant relationship between tail biting given and “fighting” (PC-A2) (F1, 85 = 0.17, *p* = 0.68) or “ear-directed manipulation” (PC-A3) (F1, 85 = 0.34, *p* = 0.56) (Annex 3).

When studying the relationship between PCA-B and tail biting received, the results of Model 4 showed that individuals that were more “explored while active” (PC-B1) received significantly more tail biting (F1, 85 = 5.29, *p* = 0.023) (Figure 3A). Individuals that were more “attacked and explored” (PC-B3) also received significantly more tail biting (F1, 85 = 11.77, *p* < 0.01) (Figure 3B). There was no significant relationship between tail biting received and “agonism” (PC-B2) (F1, 85 = 0.10, *p* = 0.75) (Annex 4).

The relationship between tail biting lesions and the predictor variables for receiving tail biting (PCA-B) were also analyzed. The results of Model 5 showed that individuals expressing more “agonism” (PC-B2) had significantly a higher risk of having a tail biting lesions [X^2^ = 5.61, *p* = 0.025; OR = 1.58, 95% CI [1.08, 2.45]] (Figure 4). There was no significant relationship between tail lesions and the behaviors “explored while active” (PC-B1) [X^2^ = 0.07, *p* = 0.78; OR = 0.95, 95% CI [0.71, 1.30]] or “attacked and explored” (PC-B3) [X^2^ = 0.68, *p* = 0.41; OR = 1.19, 95% CI [0.79, 1.83]] behaviors (Annex 5).

When studying the relationship between PCA-C and tail biting lesions, the results of Model 6 showed that there was no significant relationship between tail lesions and the behaviors “active pen and tail exploration” (PC-C1) [X^2^ = 0.01, *p* = 0.90; OR = 0.98, 95% CI [0.75, 1.29]], “non-social exploration” (PC-C2) [X^2^ = 0.75, *p* = 0.11; OR = 1.36, 95% CI [0.95, 2.07]], “ear manipulated and tail exploration” (PC-C3) [X^2^ = 0.54, *p* = 0.46; OR = 1.16, 95% CI [0.78, 1.74]], or “attacked and not tail explored” (PC-C4) [X^2^= 1.08, *p* = 0.30; OR = 1.25, 95% CI [0.82, 1.94]] (Annex 6).

## Discussion

The main objective of this study was to validate how tail biting behavior and tail damage are related to general behaviors at the individual level in pre-finishing piglets housed in a standard commercial setting. To do this, the general behaviors of individual pre-finishing piglets were related to tail biting given, tail biting received, and tail biting lesions. The results of this study demonstrated that most observations of tail biting involved “tail manipulation without reaction,” a behavior that was performed and received rather equally by all piglets and did not lead to severe wounds on the piglets' tail. When general behaviors were related to tail biting, it was detected that piglets performing more tail biting showed more “active exploration” behavior, piglets that received more tail biting were more “explored while active” and “attacked and explored,” and the piglets with higher risk of having tail biting lesions showed more “agonism” behavior. In addition, the present study discussed whether these behaviors can be related to particular types of tail biting. The results of this study indicate the presence of a pre-damage stage and probably an incipient damaging stage of the two-stage tail biting, and strongly suggest that sudden-forceful tail biting was present to a lesser extent.

### Incidence of tail biting

The results of this study showed that most of the individuals were engaged in tail biting behavior, and only two individuals acted as neutral. Some individuals acted uniquely as biters or victims; but most individuals engaged in both the acts of giving and receiving tail biting. These results are in agreement with a study that found that most pigs within a pen were involved in biting tails, being bitten, or both (5) and are differentiated by findings from another study where most individuals never performed tail biting while a few performed the majority of the tail bites, allowing the categorization of most of the piglets as only biters, victims, or neutrals (21). These discrepancies may be due to the different stage at which tail biting was observed, as well as the presence of different types of tail biting in the farms studied. In addition, on the contrary to our tail docked piglets, these studies worked with undocked tails, indicating that the motivation to perform and receive tail biting does not decrease in tail docked pigs.

During the behavioral observations conducted in this study, tail biting was observed a total of 420 times. This indicate that each pig gave and/or received an average of 2 tail bites per hour. In addition, most individuals gave few tail bites, none of them giving more than 10 during the observation period. These results are in line with another study that reported a low frequency of tail in mouth behavior (15), a behavior considered a precursor of tail biting (18). Similarly, most of these observations seen in our study were scored as “tail manipulation without reaction,” while the behavior “tail manipulation with reaction” was only observed a few times, thus both behaviors were combined into the stated “tail biting” behavior. Though relatively frequent, tail biting did not lead to wounds or severe tail lesions on any of the pigs, thus did not cause tail biting outbreaks. In the present study, no tail lesions were observed before 8 weeks of age, and at this age almost half of the piglets showed no damage on their tail. The other half only had minor incipient lesions to the tail or signs of tail manipulation, such as minor scratches or missing hair. Other studies found that almost half of the piglets studied presented tail wounds by the age of 9 weeks and tail biting outbreaks were seen as early as 2 weeks after weaning (27, 39, 40). However, these studies were carried out on undocked pigs, which may account for the higher incidence of tail wounds observed. This is consistent with the idea of docking as a tool to minimize lesions caused by tail biting and delay the onset of outbreaks, even though does not solve its causes (8).

### The relationship between general behaviors and tail biting

To validate how tail biting is related to general behavior at the individual level, piglets' general behaviors were related to tail biting given, tail biting received, and tail biting lesions. We compared the outcomes of the present study with the findings of previous literature that also related tail biting to general behaviors.

The results of this study showed that individuals performing more tail biting also engaged in more “active exploration” behavior. These results are somewhat consistent with other literature on this topic, as previous studies have shown that tail biters perform more explorative behaviors directed to enrichment devices (23) and pen mates' body parts (i.e., nosing, sniffing or tail exploration) (21–23). Piglets that performed tail biting in our study were more active, lied down less and stood more, which is consistent with studies that found that activity was higher in pens where tail biting was detected (9, 19, 20). Though not among the main loading behaviors, aggression did load into PC-A1 “active exploration,” indicating a positive correlation to giving tail biting. In contrast, the behavior “fighting” was not found to be related to giving tail biting. This is in line with previous literature that did not find a relationship between giving tail biting and aggression, though they did find that tail biters received more aggression (23). The behavior “ear-directed manipulation” was not related to giving tail biting. To conclude, our results were consistent with the findings from previous research that indicated that performing tail biting is mainly related to exploratory behaviors and enhanced activity of the biters.

The results of our study showed that individuals that received more tail biting were also more “explored while active” and “attacked and explored.” These results are partially in accordance with previous literature that characterized tail biting victims: these studies found that individuals that received more tail biting also received more tail exploration (21, 23) and nosing (22), and were more restless (i.e., spent more time standing, and less time sitting and lying down) (23). Our study also found that individuals that received more tail biting also received more ear manipulation and aggression; to our knowledge, this relationship was not previously detected. However, our study did not find a link between “agonism” (i.e., aggression) and receiving tail biting, while this relationship was detected in another study (23). To conclude, the previously established relationships between receiving tail biting and the behaviors tail exploration, pen mate exploration, and restlessness were also detected in our study. The novel finding in our study is that receiving tail biting was also related to receiving aggression and ear manipulation.

Two sets of behaviors were used in our study to explore tail lesions: receiving tail biting and having tail damage. First, we wanted to establish whether predictor variables for receiving tail biting were related to tail lesions. Our results showed that piglets with tail lesions demonstrated more “agonism.” These results are not consistent with the literature, as other studies did not find a relationship between giving or receiving aggression and having tail lesions. We also found that piglets with tail lesions received less nosing and spent more time sitting, whereas previous studies found that in pens where tail biting was detected, piglets had higher activity levels (9, 19, 20) and showed more behaviors directed toward their pen and pen mates (9), including their tail (20). Additionally, we did not detect a relationship between tail lesions and being “explored while active” or “attacked and explored” behaviors. To conclude, previous relationships between tail lesions and general behaviors were not found in this study, which on the contrary, found that tail lesions were related to giving and receiving more aggression, sitting more, and nosing less.

This study also explored whether tail lesions were related to behaviors used to predict tail biting damage. Results demonstrated that tail lesions were not related to the behaviors: “active pen and tail exploration,” “non-social exploration,” “ear manipulated and tail exploration,” and “attacked and not tail explored”. These findings are not in accordance with those from studies that found that activity levels (9, 19, 20), behavior directed toward the pen and pen mates (9), and object manipulation were higher (19) in pens with tail damage, whereas tail interest was lower (20). The lack of relationship detected between tail lesions and general behaviors in our study may be due to the absence of severe tail damage; we only observed minor scratches in a few individuals. At such a low incidence of tail biting damage, strong patterns between tail lesions and general behaviors are unlikely to be uncovered.

The marked differences between the results from our study and that of previous literature may be explained by differences in the tail biting stages observed. The minor tail damage in our study indicated an incipient stage of tail biting, whereas other studies generally observe groups of pigs where severe tail biting damage is already present, often leading to tail biting outbreaks. However, unexpected novel findings from our study regarding the relationship between tail lesions and giving and receiving aggression, sitting more, and receiving less nosing are noteworthy and should be further explored in future studies.

### Tail biting types identified

Four different types of tail biting have been identified in the literature, each with their specific motivation: two-stage, sudden-forceful, obsessive (12), and epidemic (10). Creating links between general behaviors and tail biting allows for different types of tail biting to be identified in various situations, which is necessary for implementing the appropriate measures.

The predominant form of tail biting in our study is believed to be two-stage tail biting. This tail biting type is related to the foraging and exploratory needs of the pigs (13, 14). The results of this study showed that individuals that performed more tail biting showed more “active exploration” behavior. This indicates that tail biters performed more explorative behaviors directed to their pen and pen mates' bodies and tails. Individuals that received more tail biting were also more “explored while active” and “attacked and explored”; these behaviors both suggest that individuals that received tail biting also received more exploration to their body, tail, and ears. Thus, individuals that performed tail biting did not direct their explorative behaviors exclusively to their pen mates' tails; they also focused on enrichment materials, their pen, and other body parts of their conspecifics. Similarly, individuals receiving tail exploration also received exploration to other body parts (i.e., ears). This is consistent with two-stage tail biting, where biters do not focus their explorative behaviors solely on the tail of the victim. Our results also showed that most individuals in the study engaged in both giving and receiving tail biting. This suggests that the motivation to chew or manipulate may underlie most of the animals (17).

Two-stage tail biting consists of a pre-damage stage that does not cause visible tail damage, and a damaging stage where tail damage is present (12). The behavior “tail manipulation without reaction” from our ethogram is consistent with the definition of the pre-damage stage of two-stage tail biting (12). Additionally, it is in accordance with the observations from our study that both piglets were lying down or standing still while “tail manipulation without reaction” was scored (12). Thus, the 74 individuals that gave “tail manipulation without reaction” are considered to perform the pre-damage stage of two-stage tail biting, while 71 individuals received this behavior. The manipulation of pen mates' tails that does not cause a reaction from the victim is considered a precursor to the damaging phase of two-stage tail biting. During the damaging phase, this same behavior would cause a reaction from the victim and visible tail lesions (13). This might be the case of the 8 piglets that engaged in “tail manipulation with reaction” behavior. This could indicate that the damaging stage of two-stage tail biting is already starting. Even though we did not observe any wound or severe wound on the studied animals, minor or invisible tail lesions can be painful for the animals, and they might react when bitten (24). Nevertheless, in the present study it was not possible to distinguish whether the minor lesions were provoked by the damaging stage of two-stage tail biting or by other damaging tail biting type.

Sudden-forceful tail biting was probably present in our study. This type of tail biting is considered an aggressive behavior accompanied by a reaction from the victim (12). Performing tail biting was related to “active exploration,” of which aggression was one of the loading behaviors. This suggests that, even at a low incidence, individuals that performed tail biting did perform aggressive behaviors. The animals that received more tail biting were also more “attacked and explored,” which indicates that individuals that received ear and tail exploration also received aggression. The tail lesions present in the piglets' tails were related to “agonism” indicating that these individuals were involved in both receiving and performing aggression. We observed 8 piglets engage in “tail manipulation with reaction” behavior, indicating that those individuals might have been involved in sudden-forceful tail biting. Performing tail biting was, to a certain extent, related to aggression in the studied animals and some of them reacted when being bitten, thus we suspect that sudden-forceful tail biting might be present. As only a few individuals were involved and damage was minor and infrequently, sudden-forceful tail biting was not considered the predominant tail biting type observed. Nevertheless, it was not possible to know whether the minor lesions seen were caused by this tail biting type. Thus, further studies should be conducted to differentiate between the damaging stage of two stage tail biting and sudden-forceful tail biting and classify them when seen.

Obsessive tail biting was not observed in our study. Most of the animals performed tail biting and only bite a few times. In addition, they did so without causing wounds. This is in direct contrast to obsessive tail biting, which tends to be performed by only one or a few individuals and causes wounds on the victims' tail (12). Similarly, epidemic tail biting was not observed in our study. No tail wounds or severe wounds were observed in any of the studied pens, which if appearing suddenly and unexpectedly, could indicate the appearance of this type of tail biting (10).

We can conclude that the results of our study provide evidence that two-stage tail-biting, particularly the pre-damage stage, was the prevailing type of tail biting in our groups of pre-finishing piglets. Studying the general behaviors of pre-finishing piglets and their link to tail biting in our study allowed for the detection of the tail biting types present in the animals studied: obsessive or epidemic tail biting were not observed, sudden-forceful tail biting was probably present but rarely seen, and the pre-damage stage of two-stage tail biting was predominant, being likely that the damaging stage was starting to develop.

### Tail biting preventive strategies

Since different types of tail biting have different causes, identifying the type of tail biting allows farmers to take specific measures against this detrimental behavior. The results of this study show that two-stage tail biting, and probably sudden-forceful tail biting, to a lesser extent, are most common in tail-docked weaned piglets housed under commercial conditions. Therefore, preventive strategies should be focused on avoiding the motivations for these two types of tail biting.

Our results suggest that piglets were mainly experiencing the pre-damage stage of two-stage tail biting. Also, in practice, most pigs do not have sufficient enrichment, meaning that most pigs that show tail biting are bound to present two-stage tail biting (12). If preventative measures are not taken in the pre-damaging stage, the damaging stage accompanied by severe lesions can be expected to appear (13, 14, 41). Two-stage tail biting is related to lack of exploring opportunities and should be remedied by providing suitable objects or substrates that allow pigs to manipulate, chew, and root safely (12, 41, 42). Straw or other bedding materials are optimal and meet the required characteristics. Unfortunately, farmers are reluctant to apply bedding material in commercial farms given its management limitations (42). Other materials, such as roughage, hessian sacks, compost, fresh wood, ropes, and straw supplied to the animals through feeders, racks, or cylinders may prevent the pre-damage stage from developing into the damaging stage of two-stage tail biting (12). These should be provided at multiple locations to reduce competition and should be exchanged regularly (43).

Although rarely seen in our study, sudden-forceful tail biting is related to frustration caused by the inability to access limited resources (15). This tail biting type can be minimized by improving access to resources to avoid monopolization by one or a few animals (44). Our study indicated several sources of potential monopolization: water, food, enrichment, and space. Installing additional access points for water, increasing the size of the feeding areas, increasing the number and variety of enrichment objects, and reducing stocking density may reduce competition between pigs and allow piglets to synchronize their activities within a pen, avoiding deleterious competition (45–47).

In conclusion, adopting the aforementioned measures can help prevent tail biting outbreaks. Since Directive 2008/120/EC (7) states that preventive strategies should be put in place prior to tail-docking, these strategies should be in place in any facility housing piglets at risk for tail biting (48). Strategies include an improvement of inadequate environmental conditions and management systems. Preventive measures will prevent tail biting outbreaks, avoiding its negative welfare consequences for pigs and financial burden for farmers. In this study, only tail-docked piglets were observed, so the effects of implementation of the proposed approach on piglets with intact tails should be further tested.

## Conclusion

In this explorative study, we validated how tail biting was related to general behaviors at the individual level. Results indicated that the performance of tail biting was related to exploratory behaviors and enhanced activity, which was consistent with previous literature. The reception of tail biting was related to restlessness, receiving aggression, ear manipulation, and exploring pen mates, including the exploration of their tails; this was only partially consistent with the literature. The presentation of tail lesions was related to giving and receiving aggression, sitting, and decreased pen mate exploration: this was not consistent with the literature. In addition, we were able to identify the type(s) of tail biting present in the studied animals using the behaviors linked to tail biting. The predominant type of tail biting observed was the pre-damage stage of two-stage tail biting, while it is believed that the damaging stage was at its initial phase. Sudden-forceful tail biting was probably also present to a lesser extent. Despite this, we were not able to detect whether the minor lesions present on the piglets' tail were caused by the damage stage of two-stage tail biting or by sudden-forceful tail biting. Studying tail biting in a commercial context at the individual level provides early detection and a deeper understanding of the factors that contribute to the development of this behavior. More importantly, the study and detection of tail biting types helps guide preventive actions geared toward specific causes of tail biting.

## Data availability statement

The raw data supporting the conclusions of this article will be made available by the authors, without undue reservation.

## Ethics statement

The animal study was reviewed and approved by Animal Welfare Body, Utrecht University. Written informed consent was obtained from the owners for the participation of their animals in this study.

## Author contributions

EM and ES constructed the idea and designed the study with the input from MB and LK. MB and LK conducted the data collection and its posterior analysis. MB wrote the paper with input from all the co-authors. All authors contributed to the article and approved the submitted version.

## Conflict of interest

The authors declare that the research was conducted in the absence of any commercial or financial relationships that could be construed as a potential conflict of interest.

## Publisher's note

All claims expressed in this article are solely those of the authors and do not necessarily represent those of their affiliated organizations, or those of the publisher, the editors and the reviewers. Any product that may be evaluated in this article, or claim that may be made by its manufacturer, is not guaranteed or endorsed by the publisher.
