# Supplementary material for: Individual behavioral correlates of tail biting in pre-finishing piglets
Source: Front Vet Sci. 2022 Dec 5;9:1033463. doi: 10.3389/fvets.2022.1033463 (PMC9760930; doi:10.3389/fvets.2022.1033463)
Supplement: Supplementary file 1 [file Data_Sheet_1.docx]

Supplementary Material

Annex 1. Outcome of Model 1: linear mixed effects model relating tail biting given scores to tail biting received scores. Significant model effects are shown in italic.

| **Predictors** | **Estimate** | **Std. error** | **F value** | **p value** | **Direction** |
| --- | --- | --- | --- | --- | --- |
| Tail biting received | 0.0956 | 0.122 | 0.612 | 0.436 | - |

Annex 2. Model 2: generalized linear mixed effects model relating tail biting given and received scores with tail lesions scores. Significant model effects are shown in italic.

| **Predictors** | **Estimate** | **Std. error** | **X² value** | **p value** | **Odd ratio** | **Conf. interval** | **Direction** |
| --- | --- | --- | --- | --- | --- | --- | --- |
| Tail biting given | 0.123 | 0.093 | 1.828 | 0.187 | 1.13 | [0.94, 1.37] | - |
| Tail biting received | -0.068 | 0.124 | 0.304 | 0.582 | 0.93 | [0.72, 1.19] | - |

Annex 3. Outcome of Model 3: linear mixed effects model relating the included PCs of giving tail biting (PCA-A) to tail biting given scores. Significant model effects are shown in italic.

| **Predictors** | **Estimate** | **Std. error** | **F value** | **p value** | **Direction** |
| --- | --- | --- | --- | --- | --- |
| PC-A1: Active exploration | 0.049 | 0.016 | 8.411 | *0.004* | ↑ Active exploration, ↑ TB given |
| PC-A2: Fighting | -0.010 | 0.026 | 0.168 | 0.682 | - |
| PC-A3: Ear-directed manipulation | 0.016 | 0.028 | 0.342 | 0.559 | - |

Annex 4. Model 4: linear mixed effects model relating the included PCs of receiving tail biting (PCA-B) to tail biting received scores. Significant model effects are shown in italic.

| **Predictors** | **Estimate** | **Std. error** | **F value** | **p value** | **Direction** |
| --- | --- | --- | --- | --- | --- |
| PC-B1: Explored while active | 0.038 | 0.016 | 5.290 | *0.023* | ↑ Explored while active, ↑ TB received |
| PC-B2: Agonism | 0.006 | 0.020 | 0.101 | 0.751 | - |
| PC-B3: Attacked and explored | 0.078 | 0.022 | 11.771 | *<0.001* | ↑ Attacked and explored, ↑ TB received |

Annex 5. Model 5: generalized linear mixed model relating the included PCs of receiving tail biting (PCA-B) to tail lesions scores. Significant model effects are shown in italic.

| **Predictors** | **Estimate** | **Std. error** | **X² value** | **p value** | **Odd ratio** | **Coef. interval** | **Direction** |
| --- | --- | --- | --- | --- | --- | --- | --- |
| PC-B1: Explored while active | -0.042 | 0.155 | -0.271 | 0.786 | 0.95 | [0.71, 1.30] | - |
| PC-B2: Agonism | 0.461 | 0.206 | 2.229 | *0.025* | 1.58 | [1.08, 2.45] | ↑ Agonism, ↑ tai lesions |
| PC-B3: Attacked and explored | 0.172 | 0.210 | 0.820 | 0.412 | 1.19 | [0.79, 1.83] | - |

Annex 6. Model 6: generalized linear mixed model relating the included PCs of tail biting damage (PCA-C) to tail lesions scores. Significant model effects are shown in italic.

| **Predictors** | **Estimate** | **Std. error** | **X² value** | **p value** | **Odd ratio** | **Coef. interval** | **Direction** |
| --- | --- | --- | --- | --- | --- | --- | --- |
| PC-C1: Active pen and tail exploration | -0.017 | 0.136 | -0.132 | 0.895 | 0.98 | [0.75, 1.29] | - |
| PC-C2: Non-social exploration | 0.313 | 0.196 | 1.593 | 0.111 | 1.36 | [0.95, 2.07] | - |
| PC-C3: Ear manipulated and tail exploration | 0.146 | 0.200 | 0.728 | 0.467 | 1.16 | [0.78, 1.74] | - |
| PC-C4: Attacked and not tail explored | 0.225 | 0.217 | 1.037 | 0.300 | 1.25 | [0.82, 1.94] | - |
